# Is Ambient PM_2.5_ Sulfate Harmful?

**DOI:** 10.1289/ehp.1205873

**Published:** 2012-12-03

**Authors:** Thomas Grahame, Richard Schlesinger

**Affiliations:** 1 U.S. Department of Energy, Washington, DC, E-mail: thomas.grahame@hq.doe.gov; 2 Pace University, New York, New York

Lepeule et al. (2012) associated reduced PM_2.5_ (particulate matter ≤ 2.5 µm in aero-dynamic diameter) with decreased mortality over almost four decades. Because the sulfate/PM_2.5_ ratio dropped among six localities but the PM_2.5_ mortality coefficient did not “substantially” increase, the authors concluded that sulfate must be “about as toxic” as average PM_2.5_. In a two-pollutant world, perhaps.

When a single source emits several PM_2.5_ species, and a specific species is emitted from several sources, chemical-specific associations might not reflect inherent toxicity but rather status as a marker of harmful coemissions (Grahame and Hidy 2007; Mostofsky et al. 2012). Furthermore, because total PM_2.5_ is often associated with adverse health outcomes, association of a constituent representing a large portion of total mass (e.g., sulfate) may occur unrelated to any inherent toxicity (Mostofsky et al. 2012).

Toxicological studies have not indicated adverse health effects from sulfate per se (Schlesinger and Cassee 2003). However, reducing a unit of black carbon (BC) increased life expectancy 4–9 times more than reducing a unit of PM_2.5_ (Janssen et al. 2011). Evidence from both toxicological and human panel studies with accurate subject exposure consistently has linked BC with adverse cardio-vascular health outcomes (Grahame and Schlesinger 2010). Metals and other emissions from older steel plants are particularly toxic (Dye et al. 2001).

Substantial reductions in BC and poly-cyclic aromatic hydrocarbons from diesel engines and coke ovens, various metals from steel plants, and nickel and vanadium from residual oil have occurred over the time frame examined by Lepeule et al. (2012). Sulfur was coemitted by all of these sources. Because less abundant but more toxic PM_2.5_ species were also substantially reduced over this period, changes in the sulfate/PM_2.5_ ratio as applied to mortality might reflect toxicity of coemissions, not of sulfate. Is sulfate inherently toxic or merely a coemission of harmful PM species?

Researchers must use models that include many relevant PM_2.5_ species to successfully parse adverse health effects of each (Grahame and Hidy 2007). BC (and to a lesser extent nickel) remains consistently associated with adverse health outcomes when increasingly sophisticated models—all including 18 PM_2.5_ species—are used; however, sulfate associations become negative and insignificant (Mostofsky et al. 2012).

Further, subject exposure measures must be reasonably accurate; associations found with accurate exposure may not be found when central monitor concentrations are proxies for exposure across a metropolitan area (Suh and Zanobetti 2010).

Human panel studies can examine effects of PM_2.5_ species with more accurate subject exposure. Schwartz et al. (2005) found consistent associations for measures of heart rate variability with BC, but fewer associations for PM_2.5_. In that study, the authors used an algorithm separating BC from PM_2.5_ and found no associations with the PM_2.5_ remainder (termed “secondary PM_2.5_” by the authors), which would include both secondary sulfate and its reaction products.

Any conclusions regarding sulfate toxicity are premature until consistent results from advanced models (Mostofsky et al. 2012), which are able to examine many chemical species and incorporate good exposure measures, are available and are congruent with toxicology.
